# Supplementary material for: Towards the Integrative Theory of Alzheimer’s Disease: Linking Molecular Mechanisms of Neurotoxicity, Beta-amyloid Biomarkers, and the Diagnosis
Source: Curr Alzheimer Res. 2023 Nov 8;20(6):440–52. doi: 10.2174/1567205020666230821141745 (PMC10790337; doi:10.2174/1567205020666230821141745)
Supplement: Supplementary file 1 — Supplementary material is available on the publisher’s website along with the published article. [file CAR-20-440_SD1.pdf]

Supplementary Materials

Towards the Integrative Theory of Alzheimer's Disease: Linking Molecular Mechanisms of Neurotoxicity, Beta-amyloid Biomarkers, and the Diagnosis[#]

Yaroslav I. Molkov[1], Maria V. Zaretskaia[2] and Dmitry V. Zaretsky[2,*]

[1]Department of Mathematics and Statistics and Neuroscience Institute, Georgia State University, Atlanta, GA 30303, USA; [2]Zarbio, Chapel Hill, NC 27516, USA

SUPPLEMENTAL SPREADSHEET 1

PET	CSF
14.71207	805.2
-6.19871	1198
0.6731	1616
72.71881	884
62.48001	934.1
79.66938	837.3
0.18085	1278
-2.04412	1314
-2.37885	1604
-6.2184	719.3
-0.29171	850
-2.18195	642.7
2.77993	821.2
56.9865	707.2
58.08914	627.4
67.69786	692.8
7.68274	1338
1.14566	3392
60.90481	426.5
138.7197	585.4
147.8952	469.7
63.34637	919.6
69.94252	868.6
81.91404	628.2
-6.0215	846.8

1875-5828/23 © 2023 Bentham Science Publishers

PET	CSF
17.48836	1026
57.20309	404.3
139.527	793.3
138.5031	811.3
147.9937	750.3
-5.05669	1559
4.94583	1303
19.81178	1345
2.38613	1431
2.91776	1380
1.8545	1446
92.40881	621.9
26.64421	1396
28.8298	937.9
-17.12666	1422
20.06775	1219
35.01246	1383
37.21774	1076
47.65344	802.1
72.77788	929.9
76.81433	555.8
-11.12121	2440
-5.8246	2433
-7.77391	2072
-6.71065	1898
-14.60634	1995
-6.94693	2657
3.13435	1261
8.1553	1349
37.70999	737.8
-14.27161	2064
-17.34325	2284
4.0204	1632
-3.50118	1998
31.35012	549.7
126.2362	534.8

PET	CSF
52.93036	567.5
58.60108	626.2
78.13356	646.2
12.52648	1093
28.88887	927.2
22.07613	879.1
21.82016	667.6
-15.49239	1370
-10.11702	1371
12.4871	354.4
11.26632	751.1
15.06649	859.6
29.06608	873.2
41.60861	658.8
65.55165	566.4
105.8177	517.5
121.9832	528
108.023	545.5
1.93326	2634
-17.79612	2711
0.35806	2240
-16.20123	3305
59.7431	1336
42.23869	1233
5.5956	1711
7.50553	1574
17.2127	1220
39.60023	1274
65.88638	1048
81.16582	1123
65.39413	886.6
68.9974	681.8
124.1294	443.5
137.8336	539
134.8408	489.1
-17.46139	1893

PET	CSF
-12.63734	1478
-12.73579	1646
6.87545	2559
-7.49825	690.2
-6.71065	708.3
-15.21673	418.8
45.31033	509.2
42.82939	550.7
10.59686	1910
12.32958	2079
11.60105	2221
20.18589	1405
8.07654	1192
11.8964	1207
-17.4417	1809
-6.06088	1800
-1.55187	2324
-15.17735	3020
-14.15347	2232
-8.44337	1939
-5.64739	955.7
-5.88367	1211
22.15489	737.5
5.33963	2183
53.00912	833.5
-6.06088	2093
-1.11869	1881
10.30151	1939
-15.19704	1820
18.0003	950.6
103.3761	715.5
-3.2649	1616
-6.90755	1005
2.66179	2349
3.92195	2547
-4.50537	1975

PET	CSF
-2.71358	2280
31.48795	925.1
71.89183	827.3
15.4406	1687
13.8654	1784
54.32835	1261
23.08032	858.7
18.33503	801.7
60.80636	889.9
6.22568	1432
-9.3688	917.4
1.44101	974.3
13.45191	1258
11.22694	1142
18.90604	1033
1.57884	1826
12.07361	1217
64.72467	666.1
77.05061	624.8
70.29694	582.6
-9.11283	1345
-10.62896	1685
4.49296	1415
3.37063	1352
7.48584	1272
0.31868	1301
-0.56737	1421
-3.56025	2118
74.7075	492.7
-2.96955	1644
-2.41823	1661
-7.83298	2326
-1.00055	2357
-3.16645	2545
0.37775	2315
19.39829	1358

PET	CSF
17.07487	1304
6.56041	1355
99.28062	734.6
44.24707	1156
55.05688	1225
49.87841	1057
74.7075	1099
5.35932	924.7
9.96678	957.7
14.33796	869.9
104.4985	301.5
115.8793	325.6
117.2379	340.7
137.7943	375.4
31.27136	1400
79.8269	712.2
126.8269	709.9
12.99904	1544
7.05266	1447
10.45903	1583
-1.21714	1823
7.34801	1475
-6.15933	1691
3.33125	1885
-1.47311	1726
-11.94819	1800
-0.3114	2274
-1.03993	1966
4.86707	1201
5.85157	1044
98.55209	713.7
118.0058	717.9
6.9739	1194
21.7414	879.8
109.0666	790.5
85.81266	848.4

PET	CSF
99.41845	728.8
85.41886	820.1
79.86628	916.5
86.56088	974.7
1.71667	1157
-1.78815	1440
127.2601	479.7
134.7423	451.9
89.04182	411.4
-16.02402	1775
-3.8556	1640
-13.75967	2335
74.82564	2717
36.9027	2896
84.63126	422.8
78.46829	376.1
117.6711	319.3
140.3737	365.7
20.71752	2612
31.23198	716.6
62.83443	597.3
82.1897	402
26.82142	510.8
30.0112	866.3
42.96722	691.1
71.77369	762
97.72511	678.5
15.14525	1635
5.75312	1837
10.08492	2040
5.57591	1720
2.77993	2203
-15.80743	1244
-3.95405	1338
38.26131	979.7
33.26005	922.5

PET	CSF
35.81975	734.6
48.69701	203
2.42551	998.6
80.96892	481.2
14.53486	1913
82.42598	695.5
92.36943	471.2
86.40336	560.7
83.48924	798.6
11.01035	995.8
8.98228	1034
16.8189	1918
14.53486	1882
12.11299	1676
6.36351	1332
35.89851	1264
16.2282	1163
15.10587	659
3.35094	938.8
0.37775	783.5
-4.19033	2249
-7.08476	2287
-2.24102	1816
49.52399	957.2
34.9337	769.8
-12.20416	2108
6.59979	3088
2.93745	2623
13.13687	867.5
66.0439	220.6
202.8106	1221
184.0461	921
1.91357	1099
-4.97793	874.3
5.22149	1731
2.14985	2573

PET	CSF
-0.42954	1968
4.17792	1562
-8.54182	981.6
10.79376	1554
10.55748	2144
13.33377	1626
13.62912	1867
-2.37885	2019
2.89807	1556
11.04973	1536
10.43934	1445
-4.09188	1955
43.16412	1373
58.54201	1052
71.5571	897.5
66.37863	803.1
18.25627	752.9
1.57884	1689
4.13854	1389
-5.39142	1813
18.90604	1454
36.94208	2175
63.2873	2088
31.36981	2286
25.32498	2396
77.70038	486.5
14.61362	2426
23.45443	957.1
-2.83172	2069
0.53527	2040
11.81764	840.8
-13.95657	1061
-7.53763	470.5
-1.86691	3384
-0.54768	3140
0.10209	2952

PET	CSF
64.84281	507.2
91.30617	1244
115.8202	1089
107.1369	955.8
125.0942	1098
-6.75003	1696
14.41672	1563
6.87545	1641
3.21311	1682
6.67855	1620
-15.45301	1292
-23.1518	1519
-24.1363	1677
-23.21087	1640
5.83188	3462
5.06397	2377
0.63372	1841
11.81764	1245
13.74726	1473
10.715	2385
6.9739	3555
-0.1145	3519
0.2793	964.7
-1.13838	932.1
66.91026	685.5
4.17792	1488
5.26087	1312
9.47453	1197
-1.65032	2608
2.18923	2064
14.88928	1899
10.26213	1373
4.808	1297
2.89807	1275
8.52941	1241
13.11718	1826

PET	CSF
-5.56863	2140
38.49759	1653
22.96218	2254
30.05058	1922
34.59897	318.3
25.79754	2054
34.75649	1711
40.70287	1809
6.34382	857
20.22527	640.6
19.29984	1435
6.59979	1541
15.99192	2037
19.75271	1744
60.1369	686.4
52.61532	617.5
18.72883	2696
16.2282	772.8
11.46322	955.7
17.66557	1279
10.47872	1075
11.32539	1099
22.88342	1248
5.18211	1546
7.85995	1070
24.498	499.5
71.65555	897.8
73.36858	802.8
21.30822	351.4
20.97349	347.1
26.80173	366.2
25.32498	357.1
12.44772	1739
2.40582	1704
-6.55313	1600
-2.0835	3085

PET	CSF
1.89388	1562
24.61614	1778
44.20769	539.1
55.88386	1003
-9.97919	1404
-5.52925	1295
2.76024	1020
9.76988	938.3
-6.10026	978.2
0.31868	1287
13.09749	1236
20.56	728.6
45.07405	733.9
42.84908	732.8
51.65051	638.1
52.6547	553.3
-11.37718	975.8
3.230288	1034
6.9739	1768
1.95295	1990
15.77533	1605
16.68107	1291
8.60817	1024
-2.57575	2429
6.12723	2081
5.85157	1857
3.48877	1879
-0.937608	1982
42.75063	815.6
1.496316	1095

SUPPLEMENTAL SPREADSHEET 2

PET	CSF
100.7377	294.7
-16.91007	1340
134.0532	934.2
126.6103	593.6
99.89101	751.8
92.86168	633.5
75.00285	585.9
54.99781	519.2
107.0188	448.2
1.50008	651.4
108.279	564.5
89.90818	668.9
76.32208	433.9
61.55458	749.4
110.7205	653.3
78.72426	456
52.69408	465.7
133.7381	576.4
102.8839	548.8
24.02544	834.6
96.01208	556.4
53.20602	641.6
24.81304	1694
62.77536	543.1
-10.96369	1452
-8.93562	1366
142.7758	448.6
68.78081	651.4
54.28897	308.5
81.24458	300.8
114.6388	510.7
122.0816	449.9
62.53908	741.5
54.66308	567.7
91.89687	366
77.32627	319.5

PET	CSF
133.6594	523.9
43.87296	531.8
70.9664	590.9
89.55376	652.7
80.55543	842.8
45.33002	805.3
128.7762	651
46.76739	864.5
119.837	366.3
107.2748	660.7
77.30658	461.2
83.86335	883.8
110.6811	767.6
118.1436	760
106.7234	447.8
134.8014	497
-11.39687	3114
64.17335	769.7
73.11261	313.2
150.829	541.8
26.40793	451.7
105.2073	411.7
109.6769	507
73.76238	588.2
-3.068	402.4
29.08577	611.8
19.9693	2162
90.49888	675.3
40.80132	503.1
49.77996	877.3
96.93751	596.3
99.16248	375.7
106.5069	687.3
85.87173	384.4
59.19178	652.1
75.39665	691.9

PET	CSF
160.3787	514.6
77.0703	449.4
90.36105	624.1
77.8579	532.9
126.7088	818.3
133.5609	423.6
130.7059	568.8
123.7159	761.3
90.0657	749.9
74.43184	330.9
4.47327	473.5
148.4465	664.4
133.679	652
67.77662	506.1
55.68696	816.4
100.9937	349.8
79.11806	998.2
158.9019	549.7
99.39876	471.3
52.08369	449.8
120.664	692
91.8378	750.3
82.50474	593.9
81.55962	418
45.84196	511.8
97.7448	787.8
58.99488	318.1
118.9116	653.9
90.2626	458.3
119.9748	454.5
129.5835	783.5
26.54576	731.2
90.20353	622.5
114.7176	489.4
102.8445	587.9
87.23034	493.4

PET	CSF
74.72719	437.6
104.4591	277.5
41.31326	700.9
-21.99009	553
85.65514	785.8
15.40122	1283
106.9597	255.5
144.3904	453.7
56.1989	542.4
120.6246	551.8
-8.30554	3139
126.2953	489.7
96.7603	639.1
86.56088	439.6
85.02506	382.5
92.76323	845.1
132.9899	654.9
86.14739	910.3
69.74562	579.9
84.45405	1300
1.71667	1805
-25.25863	1416
109.0075	886.2
76.83402	725.1
73.82145	338.9
103.9078	578
17.58681	2153
52.73346	675.3
23.43474	890.7
113.8315	647.1
-13.38556	1033
102.4113	787.1
86.63964	474.9
113.3787	832.7
144.6365	647.9

SUPPLEMENTAL SPREADSHEET 3

1.206051873	-50	-25	0	25	50	75	100	125	150	175	200			**T_0**	**sigma_T**	**Kexc**	**F**	**S**
0	0	2.412103746	4.824207493	4.824207493	4.824207493	8.442363112	6.030259366	7.236311239	0	0	0			**190**	**87**	**0.47**	**78**	**250**
500	0	15.67867435	37.38760807	27.73919308	36.1815562	21.70893372	8.442363112	8.442363112	0	1.206051873	0			1.89819	1.22905	0.470762	0.778371	2.49666
1000	0	20.50288184	75.98126801	12.06051873	8.442363112	2.412103746	1.206051873	1.206051873	0	0	0							
1500	0	41.00576369	55.47838617	4.824207493	0	0	0	0	0	0	0							
2000	0	25.32708934	20.50288184	4.824207493	1.206051873	0	0	0	0	0	0							
2500	0	4.824207493	9.648414986	1.206051873	1.206051873	0	0	0	0	0	0							
3000	0	6.030259366	3.61815562	0	0	0	0	0	0	0	0							
3500	0	1.206051873	1.206051873	0	0	0	0	0	0	0	0							
4000	0	0	0	0	0	0	0	0	0	0	0							
												0.103249097						
0.402017291	-50	-25	0	25	50	75	100	125	150	175	200							
0	0	0.402017291	0.402017291	0.402017291	3.216138329	6.432276657	4.422190202	2.010086455	0	0	0							
500	0	0.402017291	1.206051873	3.61815562	6.432276657	9.648414986	7.63832853	4.824207493	1.206051873	0	0							
1000	0.402017291	1.608069164	0.402017291	0	0	0.402017291	0	0	0	0	0							
1500	0	0	0.804034582	0	0	0	0	0	0	0	0							
2000	0	0	0.804034582	0	0	0	0	0	0	0	0							
2500	0	0	0	0	0	0	0	0	0	0	0							
3000	0	0.804034582	0	0	0	0	0	0	0	0	0							
3500	0	0	0	0	0	0	0	0	0	0	0							
4000	0	0	0	0	0	0	0	0	0	0	0							
	-50	-25	0	25	50	75	100	125	150	175	200							
250	7.76461E-04	0.00626854	0.029683019	0.092069191	0.205501206	0.357388183	0.517395463	0.657787562	0.765483891	0.840679673	0.438388506							
500	5.68708E-04	0.006106339	0.029097342	0.080287718	0.153335093	0.228071639	0.284557972	0.310091485	0.29826566	0.247219045	0.259570328							

1000	2.75609E-04	0.005792301	0.028026179	0.06193975	0.083526562	0.073634229	0.0336151				0.056261012							
1500	3.76773E-05	0.005257087	0.026369532	0.039629995	0.018977524													
2000		0.004120134	0.023417088															
2500			0.015272767															
3000																		
3500																		
4000																		
	-50	-25	0	25	50	75	100	125	150	175	200							
0	0	0.017640431	0.155130132	0.48117429	1.652300766	5.316020482	5.40804998	6.082165399	0	0	0							
500	0	0.098194161	1.122972925	2.517609959	6.534196044	7.151721922	4.575889	4.113850574	0.359723858	0.298158992	0							
1000	1.10800E-04	0.128073283	2.140731596	0.747025519	0.705161567	0.207215634	0.040541554	0	0	0	0							
1500	0	0.215570854	1.484141115	0.191183319	0	0	0	0	0	0	0							
2000	0	0.104351004	0.498945938	0	0	0	0	0	0	0	0							
2500	0	0	0.147357997	0	0	0	0	0	0	0	0							
3000	0	0	0	0	0	0	0	0	0	0	0							
3500	0	0	0	0	0	0	0	0	0	0	0							
4000	0	0	0	0	0	0	0	0	0	0	0							
	-50	-25	0	25	50	75	100	125	150	175	200							
0	0	2.796480606	5.071094652	4.745050494	6.388045056	9.558619287	5.044399587	3.164232295	0	0	0							
500	0	15.98249748	37.47068702	28.83973874	36.07963681	24.20562678	11.50480264	9.152720031	0.846328016	0.907892881	0							
1000	0.401906491	21.98287773	74.24255371	11.31349321	7.737201546	2.606905404	1.165510319	1.206051873	0	0	0							
1500	0	40.79019284	54.79827963	4.633024173	0	0	0	0	0	0	0							
2000	0	25.22273833	20.80797049	4.824207493	1.206051873	0	0	0	0	0	0							
2500	0	4.824207493	9.501056989	1.206051873	1.206051873	0	0	0	0	0	0							
3000	0	6.834293948	3.61815562	0	0	0	0	0	0	0	0							

3500	0	1.206051873	1.206051873	0	0	0	0	0	0	0	0							
4000	0	0	0	0	0	0	0	0	0	0	0							
	-50	-25	0	25	50	75	100	125	150	175	200							
0	0	8.428227213	0.404936769	0.014342454	1.862948866	0.36474756	0.372390163	7.966698135	0	0	0			18	23.04076722			
500	0	0.945836528	0.006330485	0.523089132	0.001877629	1.129126741	2.864739354	0.177792408	2.837497339	0.396076694	0		**P-value**					
1000	1458.24833	17.20225376	1.452913193	0.796351321	0.769429355	0.197687987	0.041951767	0	0	0	0			**0.189028391**				
1500	0	0.216710118	0.320099165	0.199072564	0	0	0	0	0	0	0							
2000	0	0.104782723	0.191024677	0	0	0	0	0	0	0	0							
2500	0	0	0.149643467	0	0	0	0	0	0	0	0							
3000	0	0	0	0	0	0	0	0	0	0	0							
3500	0	0	0	0	0	0	0	0	0	0	0							
4000	0	0	0	0	0	0	0	0	0	0	0							
